# Pineal cysts may promote pubertal development in girls with central precocious puberty: a single-center study from China

**DOI:** 10.3389/fendo.2024.1323947

**Published:** 2024-02-08

**Authors:** Shuxian Yuan, Yifan Lin, Yixuan Zhao, Mengmeng Du, Shijie Dong, Yongxing Chen, Haiyan Wei

**Affiliations:** ^1^ Department of Endocrinology and Inborn Error of Metabolism, Children’s Hospital Affiliated to Zhengzhou University, Beijing Children's Hospital Zhengzhou Hospital, Henan Children’s Hospital, Zhengzhou Children’s Hospital, Tianjian Laboratory of Advanced Biological Sciences, Zhengzhou, China; ^2^ Department of Imaging and Nuclear Medicine, Children’s Hospital Affiliated to Zhengzhou University, Beijing Children's Hospital Zhengzhou Hospital, Henan Children’s Hospital, Zhengzhou Children’s Hospital, Zhengzhou, China

**Keywords:** pineal gland, cysts, central precocious puberty, pubertal development, magnetic resonance imaging, girl, hypothalamic-pituitary-gonadal axis

## Abstract

**Introduction:**

Pineal cysts have long been considered a benign intracranial variation. However, in our clinical practice, it has been observed that some children with central precocious puberty (CPP) who have pineal cysts experience rapid progression in adolescent development. In recent years, there has been a significant increase in the prevalence of CPP in girls, leading to more diagnoses of CPP among children with pineal cysts. Despite this, there is no consensus regarding whether pineal cysts contribute to CPP as one of its organic factors. This study aimed to analyze the clinical characteristics of pineal cysts in children with CPP and explore the potential effects of pineal cysts on puberty development.

**Methods:**

This single-center study retrospectively analyzed clinical data from girls aged 3 to 10 years who underwent head/pituitary magnetic resonance imaging at the Children’s Hospital Affiliated to Zhengzhou University between 2019 and 2022. The study categorized the detection rates of pineal cysts based on systematic disease classification and compared the rates of cyst detection between girls diagnosed with CPP and those without CPP. Subsequently, CPP-diagnosed girls with pineal cysts were examined. Among CPP-diagnosed girls meeting the study’s criteria, those with pineal cysts formed the ‘cyst group,’ while those without cysts were matched in a 1:1 ratio based on age and body mass index to form the ‘non-cyst group.’ Comparative analyses were conducted to assess the clinical characteristics between these two groups. CPP-diagnosed girls with cysts were further subdivided into three groups according to cyst size (≤5 mm, 5.1–9.9 mm, and ≥10 mm) to investigate potential differences in clinical characteristics among these subgroups. The study involved an analysis of clinical data from girls diagnosed with CPP and included imaging follow-ups to explore the progression of pineal cysts over time.

**Results:**

Among the 23,245 girls who underwent head/pituitary magnetic resonance imaging scans, the detection rate of pineal cysts was 3.6% (837/23,245), with most cases being associated with endocrine diseases. The detection rate of pineal cysts in CPP patients was 6.4% (262/4099), which was significantly higher than the 3.0% (575/19,146) in patients without CPP. In comparison to the non-cyst group, the cyst group exhibited statistically significant increases in estradiol levels, peak luteinizing hormone (LH) levels, peak LH/follicle-stimulating hormone (FSH) ratios, uterine body length, and cervix length (P < 0.001). As cyst size increased, there were significant rises in LH peak, peak LH/FSH ratio, uterine body length, and cervical length (P < 0.01). Estradiol levels and left ovarian volume also showed an increasing trend (P < 0.05). Among girls who underwent follow-up imaging, 26.3% (5/19) exhibited an increase in cyst size.

**Conclusion:**

Pineal cysts are relatively common in children with CPP. They may affect the pubertal development process, with larger cysts correlating to faster pubertal development. Therefore, the authors hypothesize that pineal cysts may trigger CPP in some cases, especially when the cysts are larger than 5 mm in size, as indicated by our data.

## 1 Introduction

Central precocious puberty (CPP) is clinically common and is caused by early activation of the hypothalamic-pituitary-gonadal axis (HPGA) (1). The overall incidence is approximately 1/5000–1/10,000, and the incidence in girls is approximately 5–10 times that in boys, showing an increasing trend annually in recent years, particularly since the novel coronavirus disease-2019 (COVID-19) pandemic (2). According to a multicenter study in Henan Province, there were 5.01- and 3.14-fold increases in the number of new-onset precocious puberty cases from 2018 to 2020 and from 2019 to 2020, respectively (3). CPP can lead to premature epiphyseal closure and affect the final adult height (FAH) in children. Decreased FAH is due to accelerated bone age caused by early exposure to estrogen, which shortens the growth potential and time window in children (4). In addition, due to the premature development of secondary sexual characteristics and sexual maturity, psychological problems or social behavioral abnormalities may occur (5). The pathogenesis of CPP and related risk factors have become hot topics in research.

According to the etiology, CPP can be divided into idiopathic CPP (ICPP) of unknown etiology and organic CPP (OCPP) caused by intracranial lesions. ICPP is seen in more than 90% of cases, especially in girls (6). OCPP is reportedly more common in boys than in girls (7), and is caused by intracranial lesions such as central nervous system inflammation, malformations, tumors, and cysts. Are pineal cysts an organic factor in the pathogenesis of CPP? To the best of the authors’ knowledge, there are few relevant studies on this topic, and no unified conclusion has been reached.

The pineal gland is a pinecone-shaped neuroendocrine gland located in the epithalamus, and plays a neuroendocrine regulation function mainly through the synthesis and secretion of melatonin (8). Studies have shown that melatonin plays an important role in the initiation and development of puberty (9). Pineal cysts have long been considered benign intracranial variations, and most have no obvious clinical symptoms (10). However, whether pineal cysts affect the secretion of melatonin and thus affect the development of puberty is still unclear. In our clinical practice, it has been found that children with both CPP and a pineal cyst usually have a faster pubertal development process. Some previous studies have reported cases of precocious puberty with pineal cysts (9). Nevertheless, some scholars believe that pineal cysts are an occasional phenomenon in children with precocious puberty (11, 12), but whether there is a correlation between the two is still inconclusive.

This study aimed to investigate whether the detection rate of pineal cysts in girls with CPP is different from that in girls without CPP through retrospective analysis of pineal cyst data. Our second objective was to investigate whether there were differences in clinical characteristics between CPP patients with pineal cysts and non-cyst groups. Our third goal was to investigate whether there is a link between cyst size and pubertal development. Finally, the follow-up of the children was to understand the trend of pineal cysts and to explore further whether pineal cysts require long-term follow-up to guide the clinic.

## 2 Materials and methods

### 2.1 Study design

**Figure 1:**
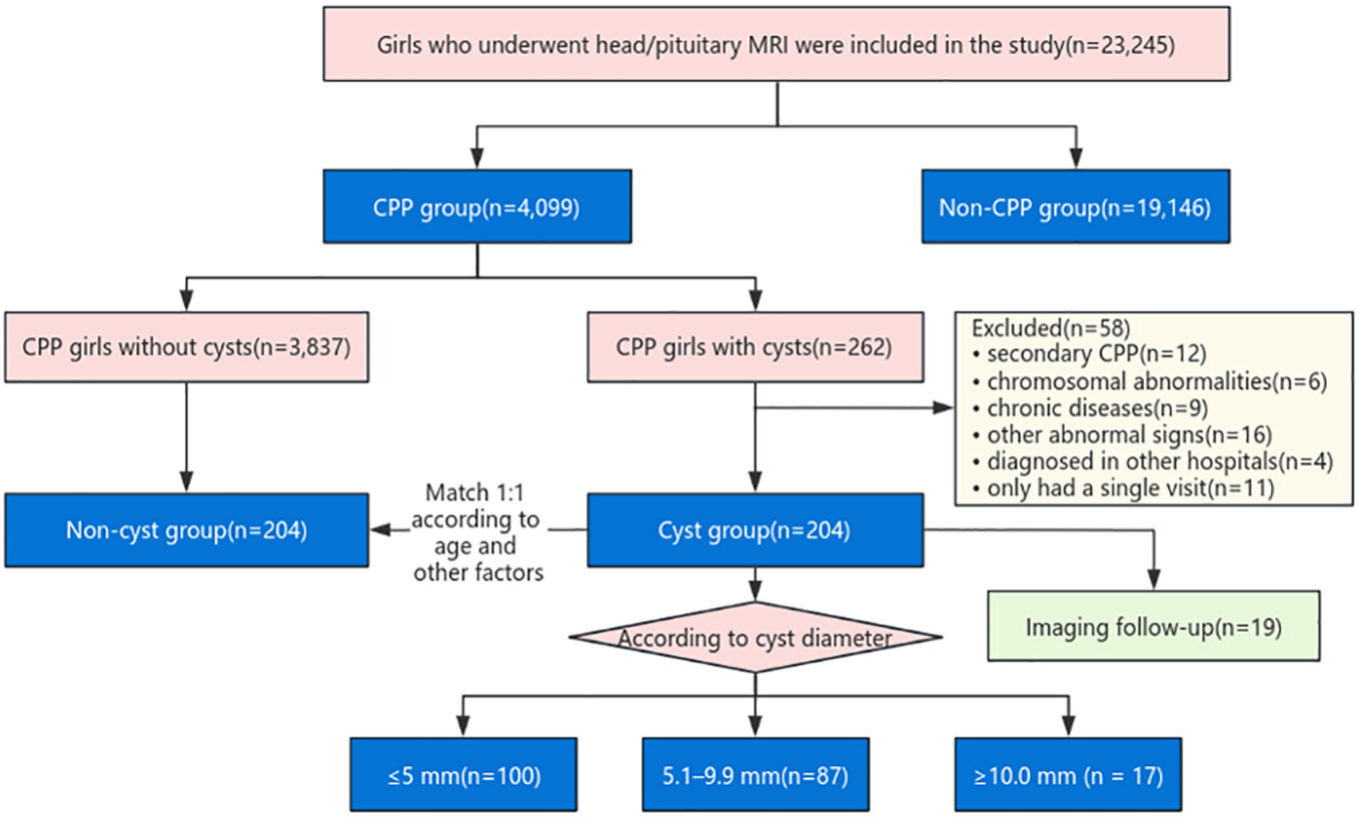
Patient selection flow diagram.

In this retrospective, single-center study, we used previous magnetic resonance imaging (MRI) data from girls aged 3–10 years who were admitted to our institution from January 2019 to June 2022 for head/pituitary MRI examination at the Children’s Hospital Affiliated to Zhengzhou University. The detection rate of pineal cysts was analyzed and compared between girls with and without CPP. Then, CPP-diagnosed girls with pineal cysts meeting the study criteria were defined as the cyst group, and CPP-diagnosed girls without cysts were defined as the non-cyst group by 1:1 matching according to age and body mass index (BMI). The clinical features between the two groups were compared per the grouping criteria in previous literature (13). Pineal cysts were grouped according to size (≤5 mm, 5.1-9.9 mm, and ≥10 mm) to explore the relationship between cyst size and pubertal development. Finally, the prognosis of girls with imaging follow-up was analyzed, and the results for pineal cysts combined with CPP were evaluated (**Figure 1**).

### 2.2 Inclusion and exclusion criteria

The study participants met the diagnostic criteria for CPP were divided into a group with a pineal cyst and a much larger non-cyst group. The criteria for the diagnosis of CPP included (1) clinical evidence of early pubertal development, defined as breast development before age 8 or menarche before age 10 in girls; (2) advanced bone age by at least 1 year compared to chronological age (CA); and (3) pubertal luteinizing hormone (LH) response >5 mIU/L at 30 min after a gonadotropin-releasing hormone analog (GnRHa) stimulation test.

Girls with both CPP and a pineal cyst were required to meet all of the following criteria: (1) all girls underwent the GnRH stimulation test and were clinically diagnosed with CPP; (2) all of them voluntarily underwent head/pituitary MRI scans; and (3) all were clinically followed up for at least 1 year.

Girls with CPP who met any of the following criteria were excluded from the study: (1) those with secondary CPP, such as congenital adrenal hyperplasia, McCuce-Albright syndrome, or ovarian tumor; (2) those with chromosomal abnormalities, endocrine or chronic diseases, or neurological diseases; (3) those whose MRI scans showed other abnormal signs, including arachnoid cysts, corpus callosum abnormalities, Rathke cysts, septal cysts, hypothalamic hamartomas, germinomas, or sellar area occupations; (4) those whose first diagnosis was made in other hospitals; and (5) those who only had a single visit.

### 2.3 Data collection

At the time of diagnosis, general data, physical measurements, pubertal stage, clinical manifestations, and laboratory and imaging findings were collected from all the girls with CPP who met the inclusion criteria. Physical measurements were interpreted using the growth charts of Chinese children and described using the standard deviation score. Pubertal development was staged according to the Marshall and Tanner criteria (14). Fasting venous blood was collected from all girls at 6 am and centrifuged within 30 min. The supernatant (3000 r/min, centrifuged for 10 min) was extracted and stored in the refrigerator at -20°C. Serum LH, FSH, and estradiol were detected using enzyme-related immunoassay. The detection kit was provided by the Beijing North Institute of Biotechnology. The GnRH stimulation test was administered via an intravenous gonadotropin injection (Fengyuan Pharmaceutical Co., Ltd., Maanshan, China) at 2.5–3.0 μg/kg (total amount not exceeding 100 μg). Blood samples were collected before (0 min) and at 30, 60, and 90 min after administration to detect LH and FSH levels, respectively. Bone age was assessed jointly by a pediatric endocrinologist and a radiologist using the Greulich-Pyle method. Two doctors performed a color ultrasound to evaluate the gonad size. Two experienced radiologists reviewed the head/pituitary MRI reports and accurately marked the pituitary height and pineal cyst diameter.

### 2.4 Ethics approval and consent to participate

All studies involving human participants were conducted in accordance with the established ethical standards and the principles outlined by the Helsinki Declaration. This study was approved by the Medical Ethics Committee of the Children’s Hospital Affiliated to Zhengzhou University on April 27, 2023 (ethics batch number: 2023-K-056). All guardians provided written informed consent.

### 2.5 Statistical analysis

Statistical analyses were performed using IBM SPSS Statistics for Windows, version 26.0. The count data are expressed as percentages (%), the comparison was performed by the *χ* ^2^ test, the measurement data conforming to the normal distribution are expressed as mean ± standard deviation (X ± S), and the comparison among multiple groups of samples was performed using one-way analysis of variance. Measurement data that did not conform to a normal distribution are expressed as median (interquartile distance) [M(Q1,Q3)]. The Mann–Whitney U test was used for comparisons between two groups, and the Kruskal–Wallis test was used for comparisons between multiple groups. P < 0.05 was considered to indicate statistical significance.

## 3 Results

### 3.1 The detection rate of pineal cyst

**Figure 2:**
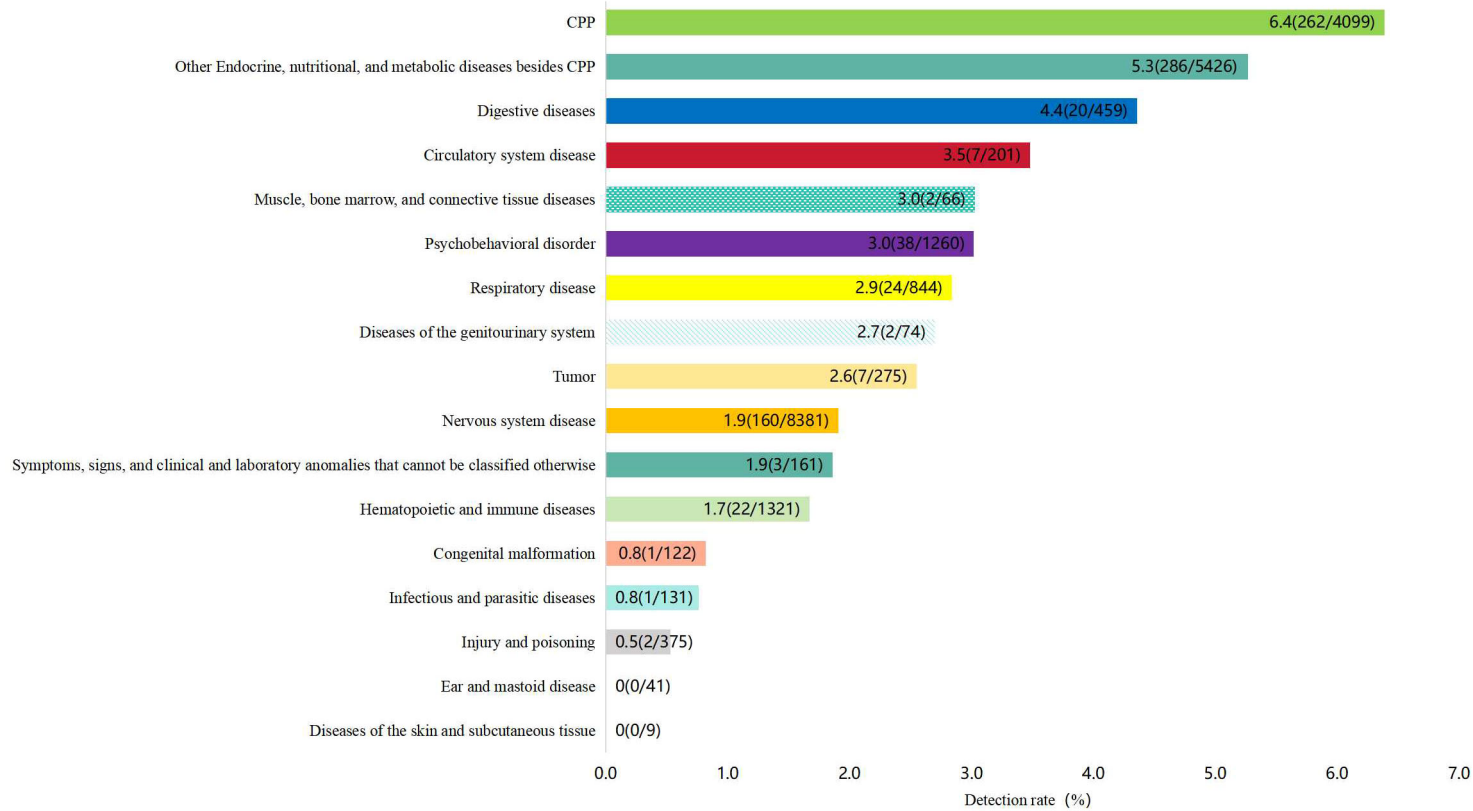
Pineal cyst detection rates in patients with different systemic diseases.

Among 23,245 girls aged 3–10 years who underwent head/pituitary MRI from January 2019 to June 2022, a total of 837 (837/23,245, 3.6%) pineal cysts were found. According to the classification of disease systems, the three systems with the highest detection rate are the endocrine system, digestive system, and circulatory system (**Table 1**, **Figure 2**). A total of 4099 children underwent MRI examinations for CPP, of whom 262 with CPP had pineal cysts (262/4099, 6.4%). The girls were divided into two groups according to whether pineal cysts accompanied CPP, and the difference in the detection rates was statistically significant (**Table 2**).

**Table 1:** Detection rate of pineal cysts.

		Total		Pineal cysts	
		N	Constituent ratio (%)	N	Detection rate (%)
Respiratory disease		844	3.6	24	2.8
Digestive diseases		459	2.0	20	4.4
Nervous system disease		8381	36.1	160	1.9
Diseases of the genitourinary system		74	0.3	2	2.7
Tumor		275	1.2	7	2.6
Diseases of the skin and subcutaneous tissue		9	0.0	0	0.0
Muscle, bone marrow, and connective tissue diseases		66	0.3	2	3.0
Endocrine, nutritional, and metabolic diseases	CPP	4099	17.6	262	6.4
	Other endocrine diseases besides CPP	5426	23.3	286	5.3
Ear and mastoid disease		41	0.2	0	0.0
Psychobehavioral disorder		1260	5.4	38	3.0
Infectious and parasitic diseases		131	0.6	1	0.8
Hematopoietic and immune diseases		1321	5.7	22	1.7
Circulatory system disease		201	0.9	7	3.5
Congenital malformation		122	0.5	1	0.8
Injury and poisoning		375	1.6	2	0.5
Symptoms, signs, and clinical and laboratory anomalies that cannot be classified otherwise		161	0.7	3	1.9

**Table 2:** Comparison of the detection rate of pineal cysts.

	N	Presence or absence of CPP	
		CPP	Non-CPP
Cyst	837	262	575
No cyst	22,408	3,837	18,571
*χ* ^2^ value		111.683	
*P-*value		<0.001	

CPP, central precocious puberty.

### 3.2 Clinical features of girls with both CPP and a pineal cyst

A total of 204 CPP-diagnosed girls with pineal cysts (cyst group) were included in the study. Among 204 girls with both CPP and a pineal cyst, the consultation age ranged from 3.52 to 9.98 years, and the mean age was 8.29 ± 0.99 years. None of the patients had symptoms of nervous system compression. Among the 204 girls, 161 were treated for “breast enlargement” as the primary complaint, 30 for “early menarche,” 8 for “early puberty” inadvertently found during the physical examination for consulting growth problems, 4 for “pubic hair growth,” and 1 for facial acne.” None of the 204 girls showed any abnormalities in the routine blood, thyroid, liver, or kidney function results. Their heights exceeded the average heights of similarly-aged children; most of them had advanced bone age and were overweight or obese according to BMI, which was in line with the general clinical characteristics of CPP (**Table 3**, group with cysts).

**Table 3:** Analysis of the clinical characteristics of girls with or without CPP [median (interquartile) [M (Q1, Q3)])].

Item	Cyst group (n = 204)	Non-cyst group (n = 204)	Z/χ^2^ value	*P-*value
Chronological age (years)	8.55 (7.80–8.86)	8.29 (7.77–8.74)	1.919	0.055
Height SDS	0.90 (0.24–1.60)	0.80 (0.02–1.52)	1.051	0.133
BMI SDS	1.07 (0.47–2.35)	1.12 (0.15–2.02)	1.195	0.232
Early menarche proportion	30 (14.7%)	17 (8.3%)	4.064	0.044*
LH base value (IU/L)	0.68(0.30–2.97)	0.69(0.32–2.60)	0.281	0.779
FSH base value (IU/L)	3.14(1.90–4.55)	2.55(1.91–4.11)	1.685	0.092
Estradiol (pg/mL)	16.12(5.00–34.20)	8.42(5.00–18.69)	4.249	<0.001*
LH peak value (IU/L)	19.00 (10.62–35.23)	14.25(9.84–27.50)	2.747	0.006*
FSH peak value (IU/L)	11.83(9.06–15.18)	11.22(8.35–14.19)	1.957	0.050
LH peak value/FSH peak value	1.78(0.98–2.53)	1.47(0.99–2.03)	2.106	<0.001*
(BA-CA)/CA	0.20(0.09–0.28)	0.18(0.08–0.27)	0.758	0.448
Uterine body length (mm)	28.90(24.53–34.80)	26.45(23.20–29.60)	4.332	<0.001*
Cervical diameter (mm)	21.85(18.28–26.10)	19.85(17.40–22.80)	3.607	<0.001*
Left ovarian volume (mL)	2.34(1.80–3.39)	2.22(1.82–2.88)	1.375	0.169
Right ovarian volume (mL)	2.44(1.81–3.48)	2.35(1.79–3.08)	1.208	0.227
Pituitary height (mm)	5.30(4.50–6.58)	5.15(4.13–6.00)	2.001	0.045*

When the hormone level was lower than the lowest detected level, the critical value was considered.

BA, bone age; CA, chronological age; CPP, central precocious puberty; BMI, body mass index; chronological age; FSH, follicle stimulating hormone; LH, luteinizing hormone; SDS, standard deviation score.

### 3.3 Comparison of clinical features between the cyst group and non-cyst group in girls with CPP

**Figure 3:**
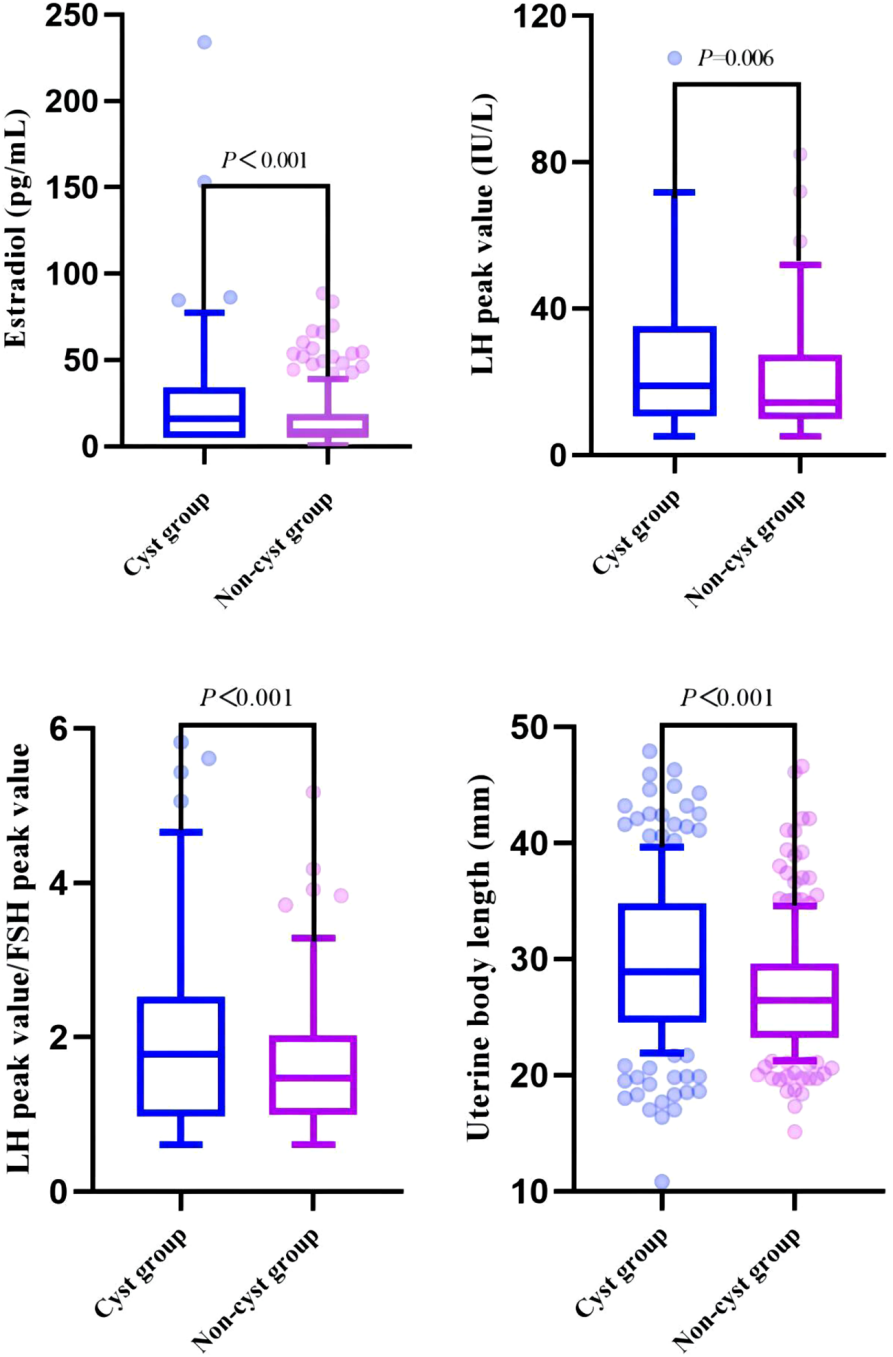
Comparison of clinical indicators related to pubertal development between cyst and non-cyst groups.

In this study, 204 girls with CPP who did not have pineal cysts on MRI examination in the hospital during the same period were randomly selected and matched according to age and BMI and included in the non-cyst group. The proportions of early menarche, estradiol level, LH peak, LH peak/FSH peak, uterine body length, cervical length, and pituitary height were significantly higher in the cyst group than in the non-cyst group, and there were significant differences between the two groups (*P* < 0.05). In particular, there were significant differences in estradiol levels, LH peak, LH peak/FSH peak, uterine body length, and cervical length (*P*<0.01) (**Table 3**, **Figure 3**).

### 3.4 Differential analysis of clinical indexes of cysts of different sizes

**Figure 4:**
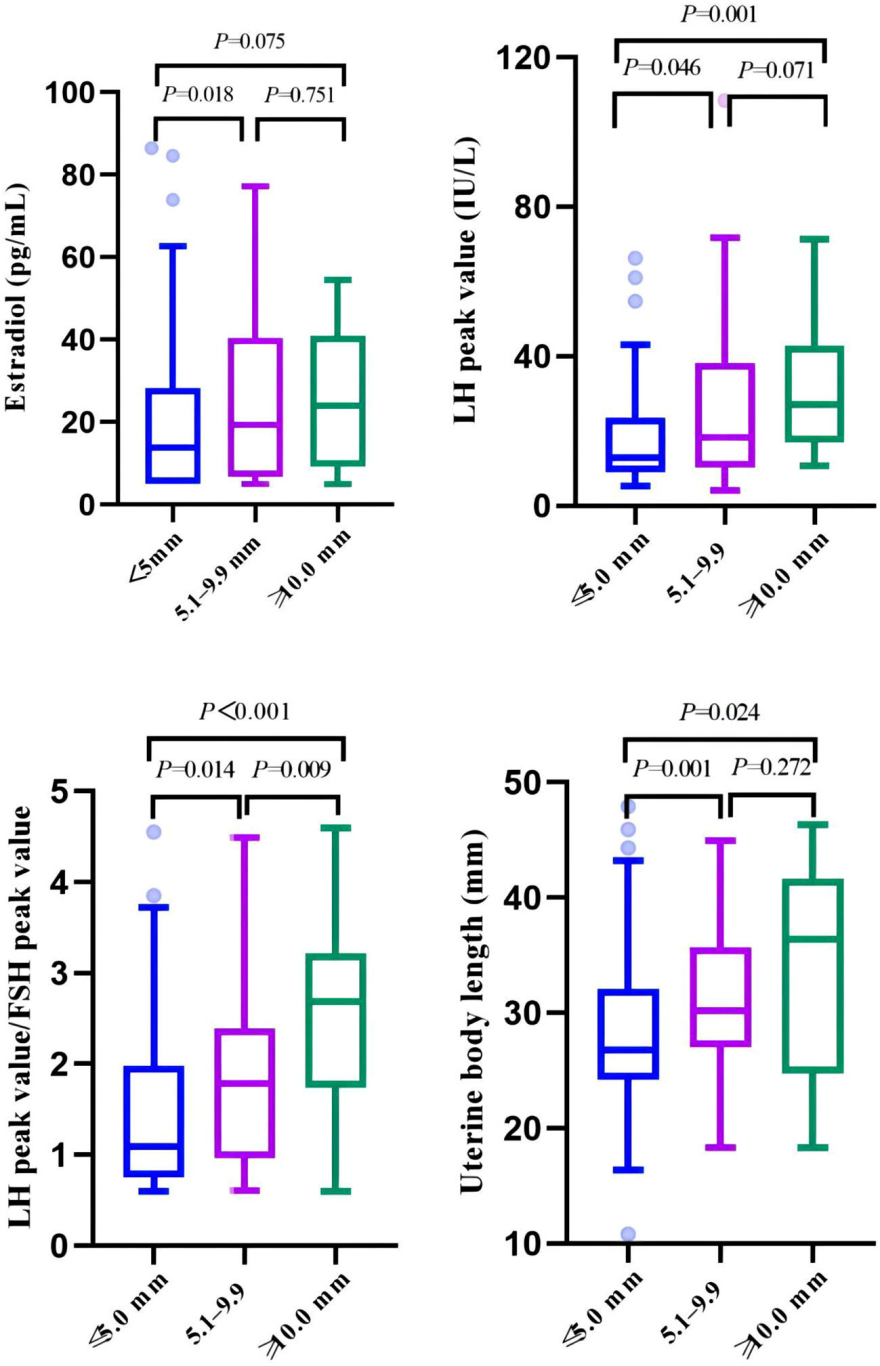
Comparison of clinical indicators related to pubertal development among three groups of cysts of different sizes.

The girls in the cyst group were divided into three groups according to the cyst diameter (≤5 mm, 5.1–9.9 mm, and ≥10 mm). There were statistically significant differences in age at first consultation, estradiol level, LH peak, LH peak/FSH peak, uterine body length, cervical length, and left ovarian volume (*P* < 0.05). In particular, there were significant differences in the LH peak, LH peak/FSH peak, uterine body length, and cervical length (*P*<0.01) (**Table 4**, **Figure 4**).

**Table 4:** Differential analysis of cysts of different sizes in the study participants (median (interquartile) [M (Q1, Q3)]).

Item	≤5.0 mm (n = 100)	5.1–9.9 mm (n = 87)	≥10.0 mm (n = 17)	H/χ^2^ value	*P-*value
Chronological age (years)	8.36(7.66–8.79)	8.65(7.99–8.87)	8.74(8.30–9.44)	6.178	0.035*
Height SDS	1.08(0.33–1.75)	0.88(0.23–1.51)	0.61(0.22–1.47)	3.050	0.218
BMI SDS	1.04(0.40–2.60)	1.33(0.41–2.36)	1.22(0.66–2.16)	0.442	0.802
Early menarche proportion	10(10.0%)	15(17.2%)	5(29.4%)	5.142	0.076
LH base value (IU/L)	0.65(0.30–2.88)	0.66(0.25–2.77)	0.93(0.48–3.45)	2.192	0.334
FSH base value (IU/L)	2.86(1.78–4.36)	3.35(2.02–4.67)	3.57(2.17–5.73)	2.134	0.344
Estradiol (pg/mL)	13.76(5.00–28.20)	19.38(6.79–40.37)	23.98(9.29–40.93)	7.019	0.030*
LH peak value (IU/L)	12.94(9.08–23.68)	18.40(10.14–38.27)	27.10(17.01–42.88)	11.403	0.003*
FSH peak value (IU/L)	12.02(9.08–23.68)	11.40(9.03–16.98)	11.90(8.59–13.93)	0.133	0.936
LH peak value/FSH peak value	1.09(0.75–1.98)	1.78(0.96–2.39)	2.69(1.74–3.22)	16.527	<0.001*
(BA-CA)/CA	0.20(0.08–0.30)	0.20(0.13–0.26)	0.24(0.07–0.32)	0.652	0.722
Uterine body length (mm)	26.80(24.23–32.10)	30.20(27.0–35.70)	36.40(24.75–41.60)	14.237	0.001*
Cervical diameter (mm)	20.30(17.53–25.08)	23.70(19.50–27.10)	23.40(19.65–30.75)	9.513	0.009*
Left ovarian volume (mL)	2.22(1.62–3.13)	2.43(1.87–3.63)	3.20(2.09–4.65)	6.169	0.046*
Right ovarian volume (mL)	2.38(1.67–3.02)	2.50(1.85–3.61)	3.17(2.27–4.02)	4.692	0.096
Pituitary height (mm)	5.25(4.50–6.28)	5.30(4.50–6.70)	5.60(4.45–6.85)	1.229	0.541

*indicates a statistically significant difference.

BA, bone age; BMI, body mass index; CA, chronological age; FSH, follicle stimulating hormone; LH, luteinizing hormone; SDS, standard deviation score.

### 3.5 Treatment and follow-up

Among the girls in the cyst group, 107 were treated with a GnRH analog (GnRHa) (107/204, 52.5%) based on height and bone age. Seventeen patients were initially treated with the Zhibai Dihuang pill (17/204, 8.3%), a Chinese herbal formula. Only 80 patients (80/204, 39.2%) were simply followed up via observation. Other treatments included vitamin D and calcium supplementation, increased outdoor activity, and dietary guidance.

In 19 girls with CPP who underwent follow-up imaging (median follow-up, 11 months; range, 3–20 months; median cyst size, 7 mm; range, 2.5%–12 mm), the cysts remained stable in 63.2% (12/19) of patients, increased in size in 26.3% (5/19), and decreased in size in 10.5% (2/19). The results of 83.3% (5/6) of the children who underwent a follow-up examination within 6 months showed that the cyst size remained stable.

## 4 Discussion

With the increasing incidence of CPP, the study of its etiology and pathogenesis has become a hot topic. Finding the potential causes of CPP is of great significance to the early intervention and prognosis improvement of CPP. Given that the incidence of CPP is significantly higher in girls than in boys, only girls with CPP were included in this study. This study found that the incidence of pineal cysts in girls with CPP was significantly higher than that in the non-CPP group, suggesting that pineal cysts may be related to CPP. By analyzing the difference in clinical data between the cyst group and the non-cyst group, we observed that sex hormone levels and gonad size were significantly higher in the cyst group than in the non-cyst group, further suggesting that cysts may promote pubertal development, and that the size of the cysts is positively correlated with the progression of puberty. Therefore, we speculate that pineal cysts may trigger CPP in some cases, especially when the cysts are larger than 5 mm as our data suggest.

Pineal cysts, which manifest as circular or oval-shaped abnormal signals in the pineal region, are usually confirmed by MRI (15). The reported prevalence of pineal cysts varies widely, with the general population at 1%–1.5%, and decreases with age (16). The prevalence of pineal cysts in children with CPP has also been reported, with detection rates ranging from 0.8% (2/251) (17) to 10.7% (6/56) (12). In this study, 4099 girls with CPP were included, and the detection rate of pineal cysts was 6.4% (262/4099), which was the largest sample size known to the author, so it was more representative. The detection rate was significantly higher than that of other systemic diseases. Therefore, we believe that pineal cysts and CPP have a certain correlation. However, besides girls with CPP, those with digestive diseases also exhibited a high detection rate (4.4%). The analysis of people with cysts found that gastrointestinal diseases mainly presented with symptoms such as vomiting. This high detection rate in individuals with digestive system diseases might be attributed to population selection, as individuals with digestive issues typically do not routinely undergo MRI unless symptoms suggest central nervous system disorders. Although there have been no studies on gastrointestinal disorders and pineal cysts, we speculate that digestive disorders may affect nutritional status and potentially delay the pubertal process by not eliciting stimulation in pineal cysts. Therefore, pineal cysts may affect the puberty process and have a specific correlation with CPP.

The clinical diagnosis and treatment decision for CPP are mainly based on auxiliary tests such as gonadotropin level (LH, FSH), gonad size, and bone age, with the peak LH value and the ratio of the LH peak value to the FSH peak value after the GnRH excitation test serving as the gold standard for early diagnosis. It is generally believed that the higher the above indicators are, the faster the pubertal development process is (18). It has been reported that CPP may be related to pineal cysts (9), but some studies have reached the opposite conclusion, suggesting that pineal cysts are an occasional phenomenon in girls with CPP (11). In this study, CPP-diagnosed girls with pineal cysts meeting the study criteria were defined as the cyst group, and CPP-diagnosed girls without cysts were defined as the non-cyst group by 1:1 matching according to age and BMI. We analyzed the difference between the clinical indicators of the cyst group and the non-cyst group in girls with CPP and found that the estradiol level, LH peak, LH peak/FSH peak, uterine body and cervix length, and pituitary height in the cyst group were significantly higher than those in the non-cyst group, suggesting that pineal cysts may be related to the pubertal development process. The reason for the difference is related to sample size and control group selection.

The clinical manifestations of pineal cysts are related to their size and can be divided into two categories: asymptomatic and symptomatic. Most pineal cysts are asymptomatic. Previous studies have reported that common clinical manifestations of pineal cysts include space-occupying headaches, vertigo, visual and eye movement disorders, and obstructive hydrocephalus (19). In some cases, ataxia, motor and sensory disorders, mood disorders, epilepsy, circadian rhythm disorders, CPP, and secondary Parkinson’s disease have been reported (20). Symptomatic cysts range in size from 7 to 45 mm, whereas asymptomatic cysts are usually <10 mm in diameter (11). According to the grouping criteria in previous literature (13), the girls were divided into three groups according to cyst size: ≤5 mm, 5.1–9.9 mm, and ≥10 mm. None of the children with CPP had neurological symptoms. However, with an increase in cyst size, sex hormone levels and gonad size further increased, suggesting that the size of pineal cysts is closely related to pubertal development and may promote premature adolescent development.

The treatment principle of pineal cysts with CPP is similar to that of ICPP. For children with rapidly progressing CPP, GnRHa is recommended for active treatment due to its impact on final adult height. Therefore, CPP-diagnosed children with pineal cysts larger than 5 mm in diameter usually show rapid pubertal progression, which can be used as an auxiliary reference indicator for GnRHa treatment. In this study, 52.5% (107/204) of the children were treated with GnRHa. Although pineal cysts are considered physiological variations of the pineal gland, some may progress to malignancy. However, whether pineal cysts change with age and whether regular follow-up is needed remain controversial. Large cysts may cause headache, nausea, vomiting, and other cranial hypertension symptoms, which can lead to pineal stroke or even death in severe cases (21, 22). In this study, the cysts of most children (63.2%) remained stable during follow-up; however, some children (26.3%) showed progressive cyst enlargement. However, none showed neurological symptoms. Therefore, we recommend active imaging follow-up when neurological symptoms are present.

The mechanism underlying pineal cysts concurrent CPP remains unclear. The pineal gland affects human reproductive function by inhibiting GnRH pulses at the gonadal level (23), which is generally believed to be related to the pineal gland’s role in melatonin secretion. Melatonin, a natural indoleamine, is synthesized from tryptophan via 5-hydroxytryptamine with the participation of vitamin D; since it is secreted mainly at night, it is known as the dark hormone (24). Melatonin receptors are widely distributed in the HPGA and inhibit the HPGA at various levels, such as in the hypothalamus, pituitary gland, and gonads, thus inhibiting the initiation of puberty (11, 25). Melatonin synthesis is regulated by sex, age, season, sunlight exposure, sleep time, and other factors (26). During the COVID-19 prevention and control period, the rapid increase in CPP incidence in children is speculated to have been related to a decrease in outdoor activities, increased exposure to artificial light, and decreased sleep time resulting from home isolation, which possibly negatively affected melatonin synthesis (3). The mechanism of action of pineal cysts leading to CPP is speculated to be as follows (27): (1) GnRH pulse suppression is eliminated due to compression of the hypothalamus by the cyst; (2) the pineal cyst adversely affects melatonin secretion, resulting in reduced inhibition of the HPGA and increased gonadotropin secretion; (3) the pineal cyst secretes gonadotropin or gonadotropin-like substances; and (4) substances conducive to the secretion of GnRH analogs are released. However, further research is needed to clarify the exact mechanism underlying this possible relationship. For children with both CPP and a pineal cyst, it is important to detect melatonin levels promptly to understand its pathogenesis.

This study had some limitations. First, since it was a retrospective study, serum melatonin concentration could not be detected in children with cysts, so it is difficult to determine the specific mechanism by which pineal cysts affect the pubertal development process. Second, the follow-up data and clinical outcomes after treatment were incomplete, and the clinical outcomes of children with both CPP and a pineal cyst could not be fully evaluated. Third, for patients with both other systemic diseases and a pineal cyst, the lack of data on pubertal development status may have led us to underestimate the detection rate of CPP with pineal cysts. Moreover, it is difficult to fully describe the clinical outcomes of pineal cysts due to the small number of children with imaging follow-up data. In addition, this was a single-center study, and the findings need to be supported by data from a larger research study; moreover, further prospective studies are needed to confirm the data and verify the practicability of our proposed conclusions. Despite these limitations, our study provides the most convincing evidence to date that pineal cysts are associated with pubertal developmental processes.

This study suggests that pineal cysts may be one of the organic lesions of CPP, accounting for 6.39% of the total number of CPP children in the same period. Pineal cysts may promote adolescent development, and the strength of the effect is positively correlated with the size of the cyst. Therefore, the authors speculate that pineal cysts may trigger CPP in some cases, especially when the cysts are larger than 5 mm as our data suggest. Imaging follow-up is recommended when neurological symptoms are present.

## Data availability statement

Due to privacy and ethical considerations, the raw patient data, including personal and medical information, are not publicly available. Requests to access these datasets should be directed to corresponding author HW, Email: haiyanwei2009@163.com.

## Ethics statement

The studies involving humans were approved by Medical Ethics Committee of the Children’s Hospital Affiliated to Zhengzhou University on April 27, 2023 (ethics batch number: 2023-K-056). The studies were conducted in accordance with the local legislation and institutional requirements. Written informed consent for participation in this study was provided by the participants’ legal guardians/next of kin.

## Author contributions

SY: Conceptualization, Formal analysis, Methodology, Writing – original draft, Writing – review & editing. YL: Formal analysis, Investigation, Writing – original draft. YZ: Formal analysis, Writing – review & editing. MD: Investigation, Writing – review & editing. SD: Investigation, Writing – review & editing. YC: Writing – review & editing. HW: Conceptualization, Methodology, Project administration, Supervision, Writing – review & editing.
